# Massive Coronary Air Embolism Treated Successfully by Simple Aspiration by Guiding Catheter

**DOI:** 10.14740/cr373w

**Published:** 2015-02-09

**Authors:** Santosh Kumar Sinha, Amit Madaan, Ramesh Thakur, Umeshwar Pandey, Kush Bhagat, Surendra Punia

**Affiliations:** a Department of Cardiology, LPS Institute of Cardiology, G.S.V.M. Medical College, G. T. Road, Kanpur, Uttar Pradesh 208002, India

**Keywords:** Aspiration catheter, Asystole, Coronary air embolism, Y-adapter

## Abstract

Coronary air embolism remains a recognized complication of coronary catheterization despite a strong emphasis on prevention. It is almost always iatrogenic. It occurs mostly when catheters used for vascular procedures have not been adequately aspirated and flushed. Current treatment consists of supportive measures with 100% oxygen and analgesia and use of aspiration catheter. Here we report a case of massive coronary air embolism of left anterior descending artery and left circumflex artery because of loose Y-adapter connection during percutaneous coronary intervention. Patient suddenly developed hypotension, chest pain, ST elevation and finally asystole. Simple vigorous aspiration was done through guiding catheter restoring the flow and finally successful intervention. Thus simple aspiration can also do the wonder as bail-out measures in the standard treatment of air embolism.

## Introduction

Coronary air embolism is a rare complication of cardiac catheterization ranging in incidence from 0.1% to 0.3%, and is almost always iatrogenic [1]. It occurs mostly when catheters used for vascular procedures have not been adequately aspirated and flushed or loose Y-adapter connection. The introduction or withdrawal of a balloon catheter or guidewire can also cause this complication. The rupture of a balloon during high inflation, and the movement of air from catheter to coronary system during intracoronary medication are rare causes [2]. Sequelae range from a clinically insignificant event to an acute coronary syndrome [3] and death [4]. Treatment has consisted mainly of supportive measures, the use of 100% oxygen (to minimize ischemia and to establish a diffusion gradient encouraging elimination of gas from the bubble), and pain relief. Mechanical interventions have been reported such as attempts at dispersal [5] or use of non-dedicated equipment for aspiration [6] or dedicated aspiration catheter.

## Case Report

**Figure 1:**
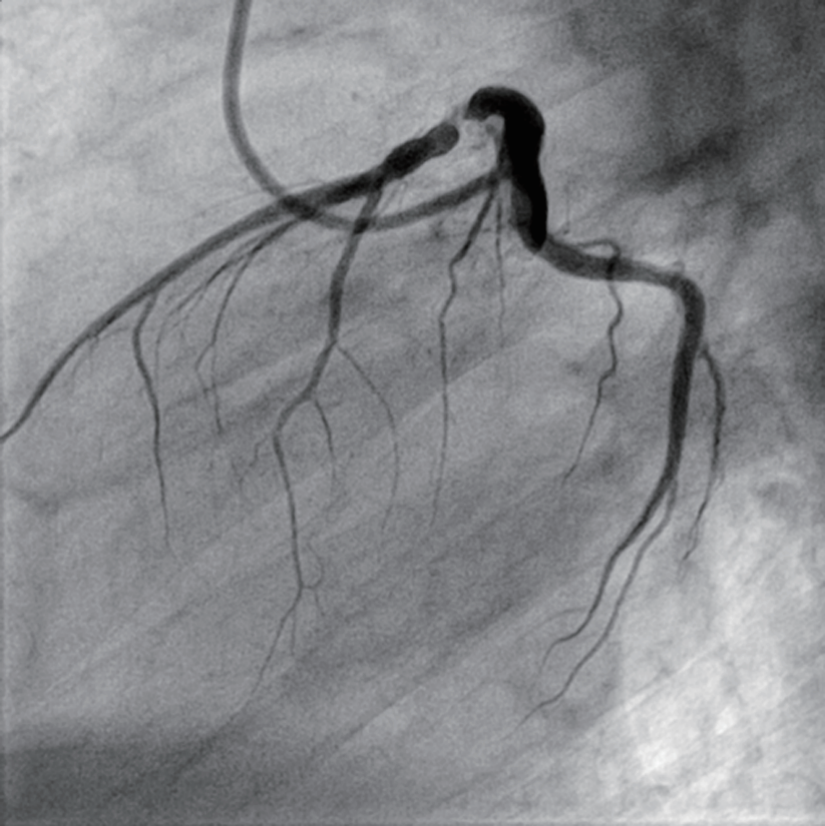
LAO view showing discrete lesion with 90% stenosis of proximal LAD (LAO: left anterior oblique view).

**Figure 2:**
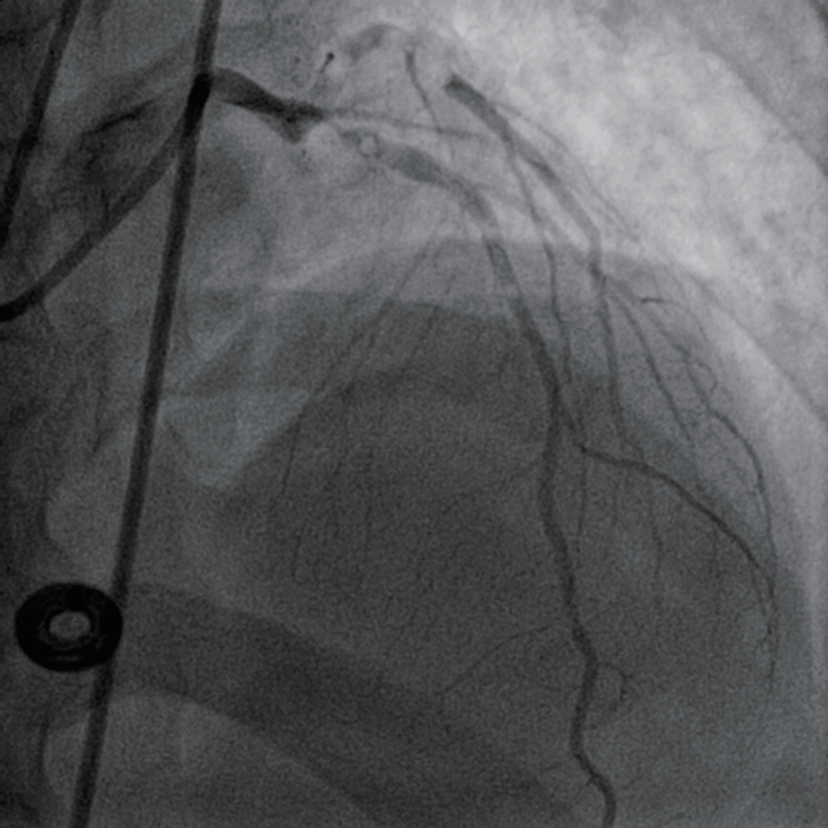
LAO view showing air bubbles in LAD and LCX (LAO: left anterior oblique view).

**Figure 3:**
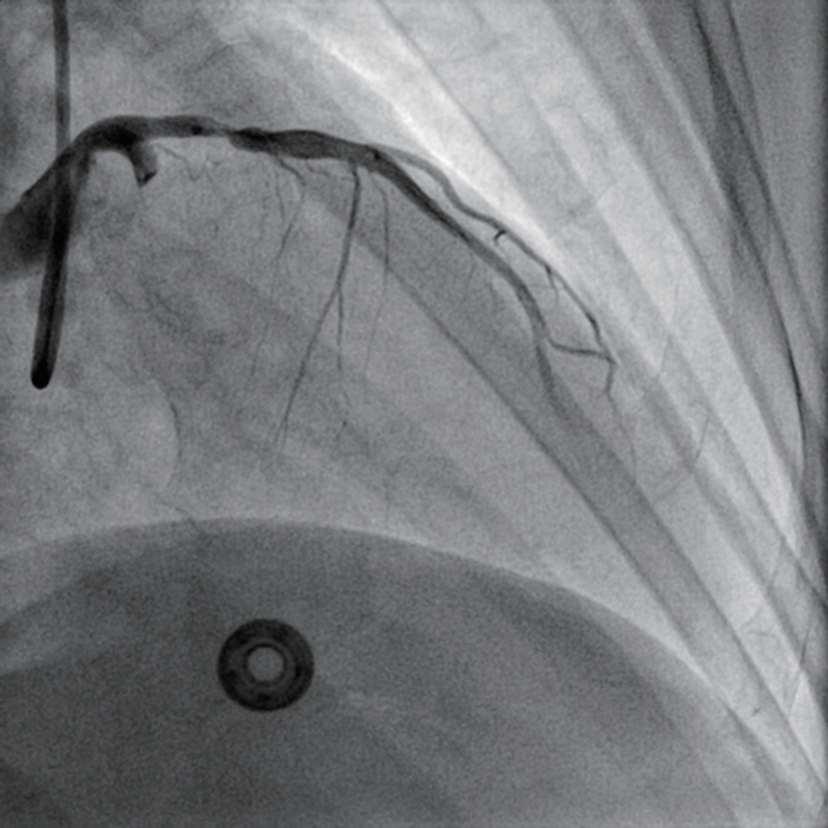
RAO view showing total occlusion of proximal LCX and distal LAD following air embolism (RAO: right anterior oblique view).

**Figure 4:**
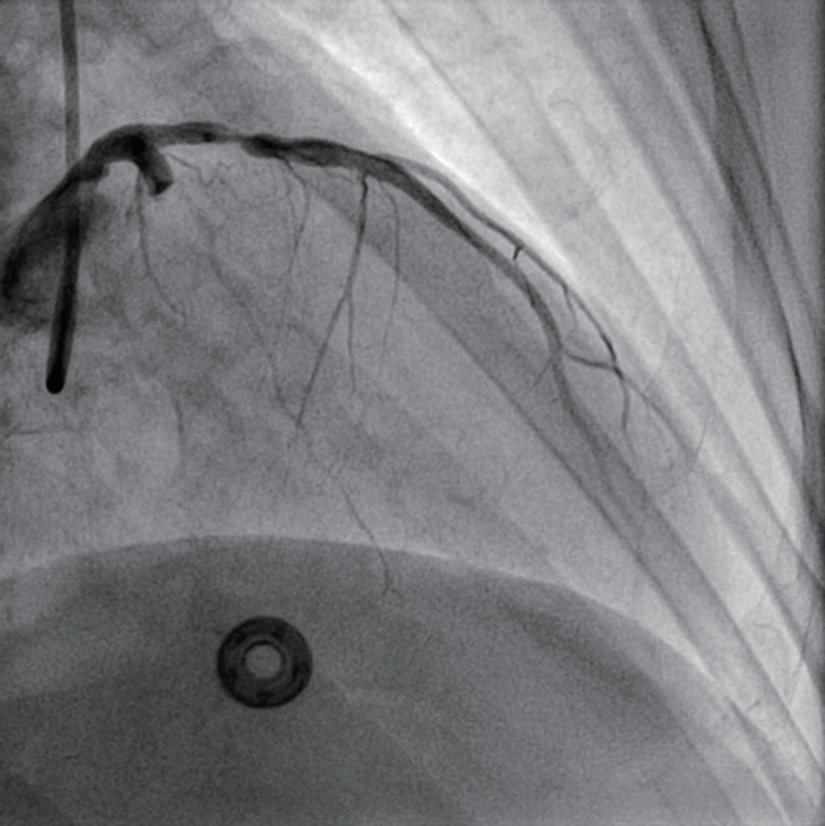
RAO cranial view of slight restoration of flow in LCX and in distal LAD (RAO: right anterior oblique view).

**Figure 5:**
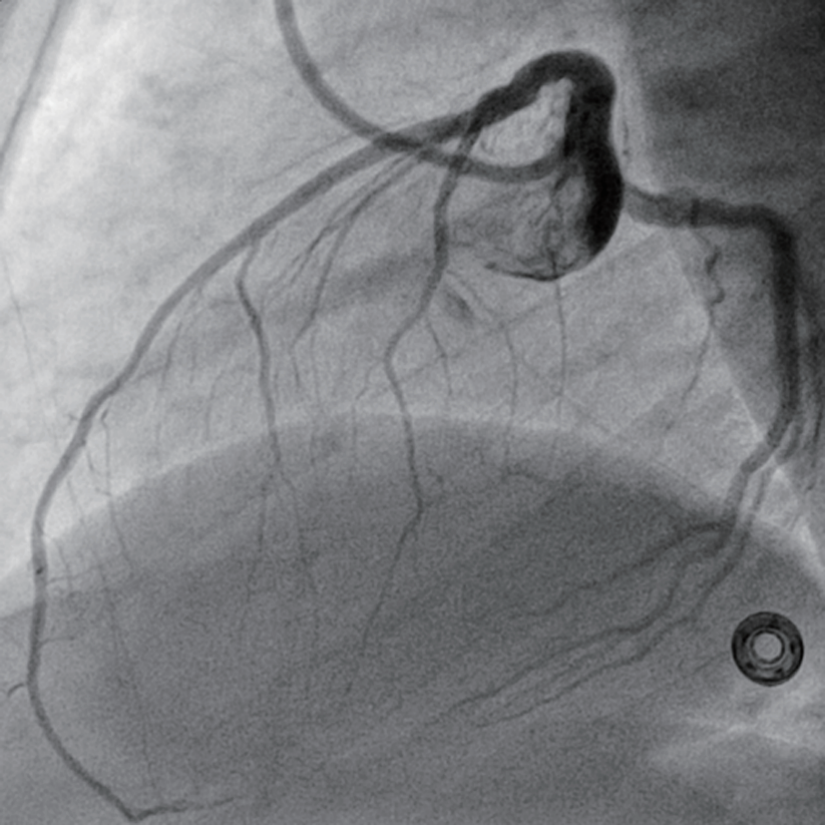
LAO view showing successful PTCA and stenting of proximal LAD and normal flowing LCX with TIMI 3 flow (LAO: left anterior oblique view).

A 38-year-old man was admitted with acute anterior wall myocardial infarction of 2 h window period. Smoking and hypertension were among the risk factors. His vitals were stable with normal biochemistry. Echocardiogram revealed anteroseptal, apical, and lateral wall hypokinesis with mild left ventricular (LV) systolic dysfunction with ejection fraction (EF) of 45%. He was thrombolyzed with single bolus of 30 mg of tenecteplase on weight-adjusted basis with successful thrombolysis. On next day coronary angiogram was performed through femoral route after proper consent. According to routine procedure, the patient received 2,500 U of heparin intra-arterially. Standard 6 F JR4 and JL4 Proflo^TM^ diagnostic catheters (Medtronic, USA) were used. Catheterization of the right coronary artery was uneventful showing a smooth, normal coronary artery. There was discrete eccentric lesion of 90% stenosis in proximal left anterior descending (LAD) artery with thrombolysis in myocardial infarction (TIMI) 2 flow with normal appearing left circumflex artery (Fig. 1). It was decided to stent LAD after proper consent. Proflo^TM^ diagnostic JL4 was exchanged with launcher JL 3.5 cm 6 F guiding catheter (Medtronic, USA) and connected via Y-adapter to manifold system and 2 - 3 mL of blood was allowed to back bleed from catheter during insertion. Further 7,000 U of heparin was given through femoral sheath. When dye was injected to check the cannulation of left system, air was introduced into both LAD and left circumflex (LCX) (Fig. 2) because of loose Y-adapter connection to manifold. Patient suddenly developed chest pain with ST-elevation in V1 to V4 and II, III, and aVF. On next shot, there was total occlusion of proximal LCX and distal LAD (Fig. 3). He then developed hypotension, bradycardia and finally asystole. Cardio-pulmonary resuscitation was started and he was given 100% oxygen, opiate analgesia and atropin. In the meantime vigorous aspiration of around 65 mL blood was done through guiding catheter after proper connection of Y-adapter. On next shot, there was little restoration of flow into both LAD and LCX (Fig. 4). All these were continued and patient gradually started showing sign of recovery. Hi-Torque BMW guidewire 0.014ʺ, 190 cm (Abott, USA) was parked beyond the lesion in distal LAD and lesion was pre-dilated with 1.25 × 8 mm balloon to 6 atm. Lesion was stented with Endeavor Sprint 2.75 × 15 mm (Zotarolimus E coronary stent system, Medtronic, USA) up to 13 atm achieving TIMI 3 flow in LAD along with normal flow into LCX (Fig. 5). Cardiac markers were assessed on next day and they ruled out any peri-procedural necrosis. The patient had an uneventful overnight stay and was discharged on the following day with aspirin 150 mg/day, prasugrel 10 mg/day, atorvastatin 80 mg/day, metoprolol 100 mg/day and ramipril 2.5 mg/day. Patient is doing excellent since then and is on regular follow-up at our institute.

## Discussion

Massive air embolism seems to be a rare event [1]. Coronary air embolism has been reported to cause chest pain, ST segment elevation, hypotension, and various arrhythmias [7]. Our case demonstrates that hemodynamic compromise can occur within no time triggering emergency and at the same time may resolve within a few minutes after prompt treatment, which includes aspiration of the air if possible, 100% oxygen administration and (often intracoronary) atropine, and epinephrine injections [7]. This case shows that guiding catheter systems can be used safely and harmlessly to resolve intracoronary air embolisms. Careful preparation of the manifold angioplasty balloons and guiding catheters is crucial to prevent this potentially life-threatening complication. Sometimes disruption or dislodgement by the guidewire and the forceful injection of saline are aimed at fragmenting the air embolus to allow dispersal or to force it distally. Main vessel patency can be restored but at the cost of damage to the distal microvasculature due to widespread, smaller embolizations. In contrast, aspiration aims at resolving the blockage by removing the air. Export aspiration catheter can also be used but it requires wiring the artery and then tracking the catheter over it which will further require more time. Our case illustrates that the guiding catheter is simple, easy to use, suitable for, and is worth considering in, the treatment of large coronary air embolus where each second counts.
